# Quantitative analysis of breast tumours aided by three-dimensional photoacoustic/ultrasound functional imaging

**DOI:** 10.1038/s41598-020-64966-6

**Published:** 2020-05-15

**Authors:** Meng Yang, Lingyi Zhao, Fang Yang, Ming Wang, Na Su, Chenyang Zhao, Yang Gui, Yao Wei, Rui Zhang, Jianchu Li, Tao Han, Xujin He, Lei Zhu, Huanwen Wu, Changhui Li, Yuxin Jiang

**Affiliations:** 1 Department of Ultrasonography, Peking Union Medical College Hospital, Chinese Academy of Medical Sciences & Peking Union Medical College, Beijing, China; 2 Department of Biomedical Engineering, College of Engineering, Peking University, Beijing, China; 3 Shenzhen Mindray Bio-Medical Electronics Co., Ltd., Shenzhen, China; 4 Department of Pathology, Molecular Pathology Research Center, Peking Union Medical College Hospital, Chinese Academy of Medical Science, Beijing, China

**Keywords:** Translational research, Breast cancer, Biomedical engineering

## Abstract

In this pilot study, we explored a quantitative method to analyse characteristics of breast tumours using 3D volumetric data obtained from a three-dimensional (3D) photoacoustic/ultrasound (PA/US) functional imaging system. Imaging results from 24 Asian patients with maximum tumour diameters less than 2 cm, including 8 benign tumours, 16 T1 stage invasive breast cancers (IBCs), and 22 normal breasts, were analysed. We found that the volumetric mean oxygenation saturation (SO_2_) in tumour regions of T1 stage IBCs was 7.7% lower than that of benign tumours (P = 0.016) and 3.9% lower than that of healthy breasts (P = 0.010). The volumetric mean SO_2_ in tumour surrounding regions of T1 stage IBCs was 4.9% lower than that of benign tumours (P = 0.009). For differentiating T1 stage IBCs and benign tumours, with a cut-off SO_2_ value of 78.2% inside tumours, we obtained a sensitivity of 100%, a specificity of 62.5%, and an AUC of 0.81; with a cut-off SO_2_ value of 77.9% in regions surrounding tumours, we obtained a sensitivity of 100%, a specificity of 75% and an AUC of 0.84. Our preliminary results demonstrate that 3D PA/US functional imaging has the potential to provide valuable quantitative physiological information that may be useful for the detection and evaluation of breast tumours.

## Introduction

Breast cancer has become the most common cancer among the female population worldwide and accounted for approximately 14.6% of all new cancer cases in the United States in 2016^1^. Early detection of breast cancer (T1 stage) is of vital importance since it contributes to lower mortality and lower burdens on society as a whole^2^. X-ray mammography and breast ultrasonography (US) are the two most widely used modalities for breast cancer screening^3^. Mammography has superior sensitivity to microcalcifications^4^, and US is effective for detecting invasive cancer in dense breasts^5^. Although X-ray and US imaging have played important roles in clinical diagnosis of breast cancers, new methods and technologies are desired to further improve the diagnostic accuracy to reduce the unnecessary biopsy of benign lesions^6^. As a novel imaging modality, photoacoustic (PA) breast imaging has received much attention in the past decade. PA imaging combines the advantages of both high-resolution ultrasonic anatomic detection and the high contrast of optically absorbing tissues such as vascular networks ^7–9^, which are closely related to tumour angiogenesis and metastasis ^10,11^. Furthermore, with two or more optical wavelengths, PA imaging can provide functional information such as the oxygen saturation (SO_2_), which is important for revealing tumour metabolism and therapeutic response^12^. Owing to these unique characteristics, PA breast imaging has made significant progresses ^13–17^.

The integration of PA imaging with traditional handheld US systems has gained much interest in clinical studies since the operators are familiar with the imaging procedure and can obtain both functional and morphological information ^18–22^. Two-dimensional (2D) PA/US imaging based on handheld US systems has showed promising progress in clinical breast imaging ^13,14^. A multi-central study that enrolled 2105 subjects showed that PA/US increases the specificity of breast mass assessment compared with US alone^13^. Another study employing multispectral PA/US imaging resolved breast cancer features with high resolution and revealed patterns not offered by other radiologic modalities^14^.

Although these pioneer PA clinical studies have obtained promising results, they mostly rely on 2D imaging and have not yet provided a robust quantitative analysis method for early breast cancer detection. Instead, with the aid of 3D anatomy information of tumours gained from 3D US imaging, 3D PA/US imaging can provide volumetric functional information such as volumetric mean SO_2_ value in both tumour regions and tumour surrounding regions. Without the need to select 2D B-scan slices, variance can be reduced, and quantitative analysis can then be carried out with more stability. Furthermore, based on 3D PA/US imaging, 3D morphological distribution of vessels can be reconstructed, which may allow a more comprehensive assessment of blood supply in lesions. The feasibility of 3D PA/US imaging has been verified by either combining a traditional handheld US system with a scanning linear probe or by utilizing a 2D array ^23–25^, although few systems have been applied to clinical trials of breast imaging.

In this study, we performed a pilot clinical study of a self-developed 3D PA/US imaging system. The purpose is to explore the potential value of quantitative analysis with 3D PA/US functional imaging in patients with breast tumours. Specifically, we aimed to explore the potential of 3D PA/US functional imaging to aid traditional US imaging in achieving a higher diagnostic accuracy. The presented imaging method can provide complementary functional information, such as SO_2_, for US imaging. In this work, we did a pilot study to assess the potential value of the volumetric mean SO_2_ in differentiating benign breast tumours from malignant ones based on 3D PA/US functional imaging.

## Materials and Methods

Our study protocol was approved by the institutional ethics committee of Peking Union Medical College Hospital. All research was performed in accordance with relevant regulations. Written informed consent was obtained from all participants.

### PA/US dual mode functional 3D imaging system

The dual-modality system (see Supplementary Fig. S1) in this study is based on a high-end clinical US machine (Resona 7, Mindray Bio-Medical Electronics Co., Ltd.)^20^, which is capable of performing parallel data acquisition required by PA imaging. Results of PA imaging were reconstructed online with the delay and sum algorithm. The clinical linear probe (L9-3U, Mindray Bio-Medical Electronics Co., Ltd.) has 192 elements, each element has a size of 0.2 mm and the central frequency is 5.8 MHz. The laser source is an OPO tunable laser (Spitlight 600-OPO, Innolas laser GmbH), which generates 700–850 nm laser pulses at 10 Hz. In our study, 750 nm and 830 nm were used for PA functional imaging. We chose these two wavelengths for two reasons. First, deHb has stronger absorption at 750 nm, and Hb has stronger absorption at 830 nm^26^. Second, the pulse energy of the OPO laser is still high at these two wavelengths. Good signal-to-noise can be achieved by comprehensively considering the above two factors. A time division multiplexing approach was employed to achieve PA/US real time imaging with two wavelengths and SO_2_ mapping at a 5 Hz frame rate. Before calculating SO_2_, PA signals of different wavelengths were normalized by laser energies recorded in real time. By scanning the probe across the breast skin surface (see Supplementary Movie S1), the system could perform local 3D dual-modal functional imaging (see Supplementary Fig. S2). During 3D image acquisition, the electric motor moved at a steady velocity (1 mm/s), while a group of 2D US images and PA images at two wavelengths was acquired at 0.2 mm step intervals with a total scanning length of 4 cm and total scanning time of 40 seconds. 3D imaging results were downloaded for further data analysis.

To render 3D PA/US images (see Supplementary Fig. S2), we imported the 2D SO_2_ map into Amira (version 6.0, Visage Imaging) and obtained vessel networks by extracting the surface of the SO_2_ map using pixels with SO_2_ values larger than 40% and less than 100%. We chose 40% as a lower bound because previous studies have shown that SO_2_ values in breast tumours normally do not go below 40%^17,27,28^. Additionally, because the patients we enrolled were all at Stage 1, abnormal SO_2_ values caused by necrosis would not be present in our study. Based on the above reasons, SO_2_ values below 40% were not employed, as these were likely caused by system noise and unwanted PA signals from the probe itself. After vessels networks were obtained, the surface of the tumour region identified in B-mode was then coregistered with the vessel map at a certain degree of transparency.

### Quantification analysis

In the near-infrared range, the dominant endogenous photoabsorber is haemoglobin, which can be either oxy-haemoglobin (Hb) or deoxy-haemoglobin (deHb)^29^. PA signal with optical wavelength ${\lambda }_{i}$ at the spatial position *r* can then be described according to Eq. (1)^30^. Γ is the Grüneisen parameter of the tissue. $F({\lambda }_{i},r)$ is the optical fluence with optical wavelength ${\lambda }_{i}$ at the spatial position $r$. $C(Hb,r)$ and $C(deHb,r)$ are the concentrations of Hb and deHb at r, respectively. ${\varepsilon }_{Hb}({\lambda }_{i})$ and ${\varepsilon }_{deHb}({\lambda }_{i})$ are the known molar extinction coefficients of Hb and deHb, respectively, at wavelength ${\lambda }_{i}$.

1 $${\boldsymbol{PA}}({{\boldsymbol{\lambda }}}_{{\boldsymbol{i}}},{\boldsymbol{r}})={\boldsymbol{\Gamma }}{\boldsymbol{F}}({{\boldsymbol{\lambda }}}_{{\boldsymbol{i}}},{\boldsymbol{r}})({\boldsymbol{C}}({\boldsymbol{Hb}},{\boldsymbol{r}}){{\boldsymbol{\varepsilon }}}_{{\boldsymbol{Hb}}}({{\boldsymbol{\lambda }}}_{{\boldsymbol{i}}})+{\boldsymbol{C}}({\boldsymbol{deHb}},{\boldsymbol{r}}){{\boldsymbol{\varepsilon }}}_{{\boldsymbol{deHb}}}({{\boldsymbol{\lambda }}}_{{\boldsymbol{i}}}))$$

Because both the absorption coefficient ${\mu }_{a}(\lambda )$ and the reduced scattering coefficient ${\mu {\prime} }_{s}$ of the background breast tissue at 750 nm and 830 nm are similar ^31,32^, the optical fluence $F({\lambda }_{750nm},r)$ and $F({\lambda }_{830nm},r)$ were assumed to be approximately the same in our study after normalization with laser illumination power at each wavelength. The definition of oxygenation saturation (SO_2_) is as follows,

2 $${\boldsymbol{S}}{{\boldsymbol{O}}}_{2}({\boldsymbol{r}})={\boldsymbol{C}}({\boldsymbol{Hb}},{\boldsymbol{r}})/({\boldsymbol{C}}({\boldsymbol{Hb}},{\boldsymbol{r}})+{\boldsymbol{C}}({\boldsymbol{deHb}},{\boldsymbol{r}}))$$

Then, the SO_2_ at each pixel can be derived from two PA values at two different wavelengths, as shown in Eq. (3)^33^. Any pixels with negative calculated SO_2_ values were removed from the latter analysis.

3 $$S{O}_{2}(r)=\frac{PA({\lambda }_{750nm},r){\varepsilon }_{deHb}({\lambda }_{830nm},r)-PA({\lambda }_{830nm},r){\varepsilon }_{deHb}({\lambda }_{750nm})}{PA({\lambda }_{750nm},r)({\varepsilon }_{deHb}({\lambda }_{830nm},r)-{\varepsilon }_{Hb}({\lambda }_{830nm},r))+PA({\lambda }_{830nm},r)({\varepsilon }_{Hb}({\lambda }_{750nm}))-{\varepsilon }_{deHb}({\lambda }_{750nm}))}$$

3D quantification analysis was then carried out in both tumour and tumour surrounding regions. The 3D tumour outer boundary was rendered based on series of 2D tumour contours marked by experienced physicians. The principle of delineation is to identify the tumour boundary where there is the highest grey scale contrast. After obtaining the 3D tumour outer boundary, we calculated the least volume ellipse (LVE) enclosing the 3D tumour region automatically in MATLAB (MathWorks, Inc.)^34^. This method has been verified to semi-automatically estimate tumour volume in CT ^35^. The analysis process was repeatable due to the uniqueness of LVE enclosing the 3D tumour region. By extending each of three axis lengths of LVE 1.2 times, we obtained an extension ellipse. We defined the region inside the extension ellipse excluding the tumour region as the surrounding region (see Supplementary Fig. S3).

After labelling regions of interest, we calculated SO_2_ pixel average values in both regions, and a lower threshold of 40% was set to minimize artifacts. Similarly, we also calculated SO_2_ pixel average values in symmetrical locations in lateral normal breasts.

### Patients and imaging procedure

Forty-five patients with breast tumours smaller than 2 cm and receiving a BIRADS rating from 3 to 5 with indication for surgery or biopsy were consecutively recruited from the outpatient and inpatient breast surgery department of Peking Union Medical College Hospital from November 2017 to January 2018. All patients went through imaging examinations via US and/or X-ray mammography, as well as MRI, by experienced radiologists. A conventional US examination was performed on all patients by 3 radiologists with more than 10 years of experience with breast US diagnosis. Only patients with clear tumour boundaries under US imaging were enrolled in our studies. 2D and 3D PA/US dual modality images were examined after conventional US. Biopsy and surgical resection were performed on all of the enrolled patients (patients graded with BIRADS 3 were all found to have an increasing tumour size in follow-up visits before biopsy). Pathological diagnoses were confirmed afterwards.

Of these forty-five patients, two patients were not imaged successfully due to system malfunctions; for another two patients, from US images, the distal margin depth of the breast lesions was more than 3.5 cm from the skin, which was beyond the effective imaging depth of the current system due to the strong light attenuation in tissue. Of the remaining 41 patients, 17 either had an intraductal lesion or distal metastases, 16 were T1 stage invasive breast cancers (IBCs) without distal metastases, and 8 were either breast fibroma or breast adenosis. The results for intraductal lesions will be investigated in a different study to reduce confounding caused by the inherent pathogenesis difference between ductal carcinoma *in situ* (DCIS) and invasive breast cancer^36^. Results of lesions with distal metastases are also not included due to the focus on early breast cancer detection of this study.

The results for the measurements on the remaining 16 T1 stage IBCs, 8 benign lesions (six fibromas and two breast adenosis) as well as 22 contralateral healthy breasts (14 from patients with T1 stage IBCs, 8 from patients with benign lesions) were studied in this work. Two contralateral breasts were excluded because there were lesions inside. Detailed information on these 24 patients is listed in Supplementary Table S1. Each image of lesions (benign, malignant or healthy) was treated as one sample. Samples were grouped into three categories: benign group (either fibromas or breast adenosis, n = 8), malignant group (T1 stage IBCs, n = 16) and normal group (contralateral healthy breasts, n = 22).

To further reduce confounding effects of tumour sizes, we performed subgroup analyses based on results from tumours with similar sizes and similar numbers (8 malignant tumours and 8 benign tumours with similar sizes in each subgroup analysis). Information on tumour sizes and corresponding tumour numbers in the subgroups is summarized in Supplementary Table S2. To match malignant tumours and benign tumours with a size ranging from 1.6 to 2 cm, two out of ten malignant tumours with a size from 1.6 to 2 cm were included in each subgroup analysis. We repeated this procedure 45 times to cover all of the combinations.

### Statistical analysis

We used the nonparametric two-tailed Mann-Whitney U-test to calculate the statistical significance between two groups, and a P value of 0.05 was considered to indicate 95% statistical significance. The Hodges-Lehmann estimator was used to determine the difference between two groups and the 95% confidence interval. The statistical analysis was performed with MATLAB (MathWorks, Inc.).

## Results

### Statistics

**Figure 1:**
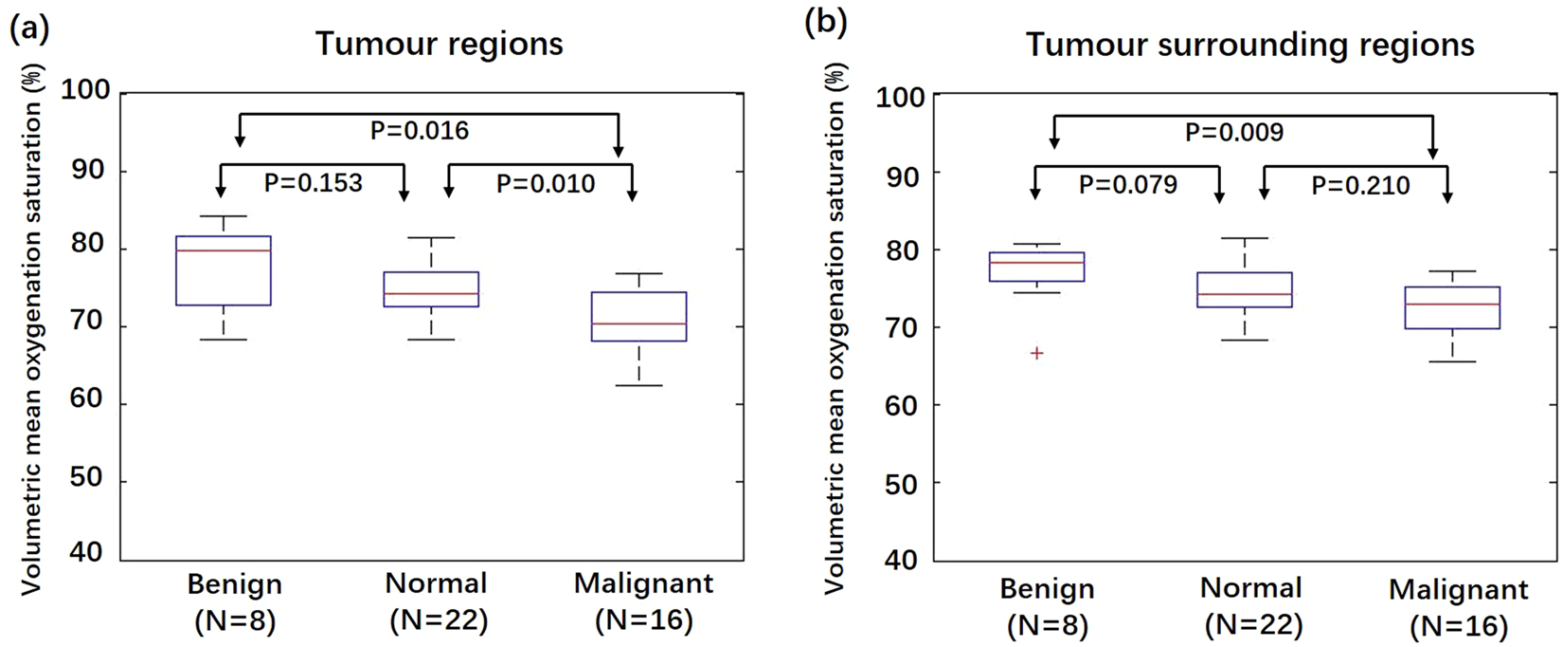
Box plots of volumetric mean oxygenation saturation of different imaging groups in tumour regions and tumour surrounding regions. (**a**) Volumetric mean SO_2_ value of the benign, normal and malignant groups in tumour regions. (**b**) Volumetric mean SO_2_ value of the benign, normal, and malignant imaging groups in tumour surrounding regions.

**Figure 2:**
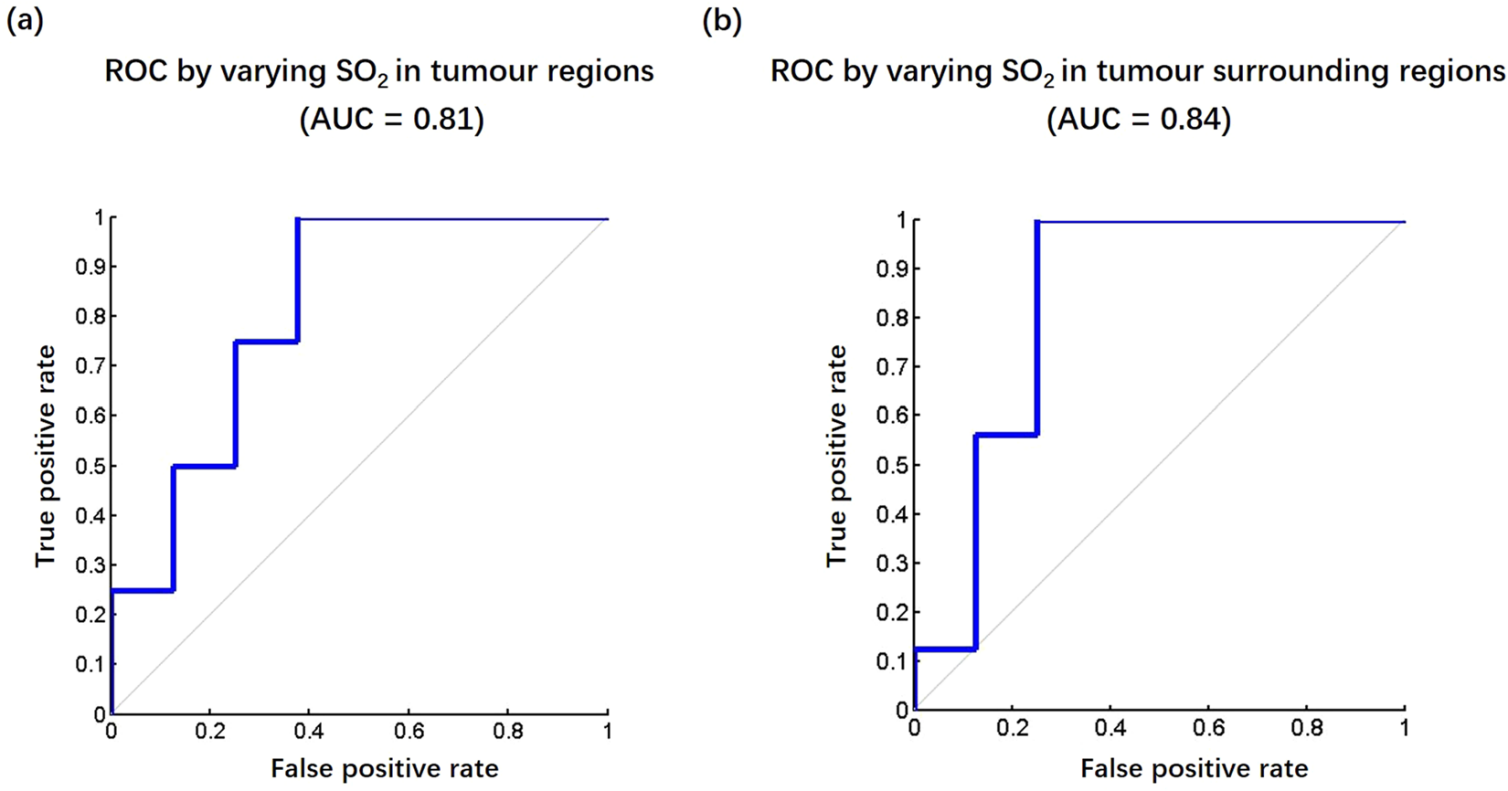
ROC for differentiating malignant tumours from benign tumours by varying SO_2_ threshold in both (**a**) tumour regions and (**b**) tumour surrounding regions.

Results from 24 patients (16 T1 stage IBCs, 8 benign lesions and 22 contralateral healthy breasts) in the imaging study are shown in Figs. 1–2. There were 22 contralateral healthy breasts instead of 24 because all but 2 patients had lesions (either benign or malignant) in one breast only while the other breast was healthy. As shown in Fig. 1a, in tumour regions, the SO_2_ volumetric mean value of the malignant group was 7.7% lower (95% confidence interval: 2.1%, 12.4%) than that of the benign groups (P = 0.016) and was 3.9% lower (95% confidential interval: 2.2%, 5.5%) than the normal group (P = 0.010). In tumour surrounding regions (Fig. 1b), the SO_2_ volumetric mean value of the malignant group was 4.9% lower (95% confidence interval: 1.6%, 8.4%) than that of the benign group (P = 0.009). Differences in SO_2_ volumetric mean values between the benign group and normal group were not significant at the 95% level.

From all 45 subgroup analyses with control of tumour size distribution, SO_2_ volumetric mean values in both tumour regions and tumour surrounding regions were significantly lower in malignant tumours than in benign tumours. In tumour regions, the SO_2_ volumetric mean value of the malignant group was at least 7.0% lower (95% confidence interval: 1.4%, 14.0%) than that of the benign group (0.005 ≤ P ≤ 0.028). In tumour surrounding regions, the SO_2_ volumetric mean value of the malignant group was at least 4.0% lower (95% confidence interval: 0.5%, 9.3%) than that of the benign group (0.010 ≤ P ≤ 0.028).

Receiver operating characteristic (ROC) curves for differentiating malignant tumours from benign tumours were plotted by varying the SO_2_ thresholds both inside tumours (Fig. 2a) and in tumour surrounding regions (Fig. 2b). With a cut-off value of 78.2% for the SO_2_ value inside tumours, we obtained a sensitivity of 100% and a specificity of 62.5%. The area under the ROC curve (AUC) was 0.81. With a cut-off value of 77.9% for the SO_2_ value in tumour surrounding regions, we obtained a sensitivity of 100%, a specificity of 75% and an AUC of 0.84.

Similarly, for all 45 subgroup analyses with control of tumour size distribution, with a cut-off value of 78.2% for the SO_2_ value inside tumours, we obtained an average sensitivity of 100%, an average specificity of 62.5% and an average AUC of 0.86. With a cut-off value of 77.9% for the SO_2_ value in tumour surrounding regions, we obtained an average sensitivity of 100%, an average specificity of 62.5% and an average AUC of 0.85.

### PA/US images of breast tumours

**Figure 3:**
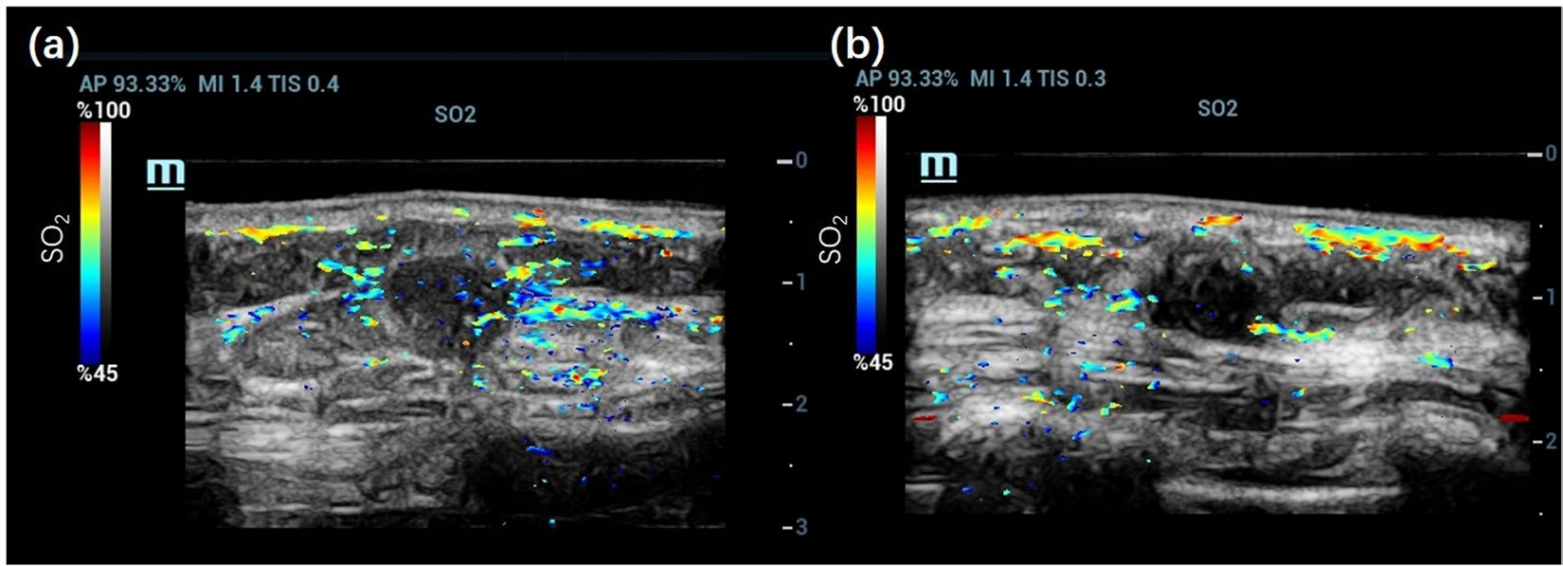
2D SO_2_/US imaging results of (**a**) an invasive breast cancer and (**b**) a breast fibroadenoma.

**Figure 4:**
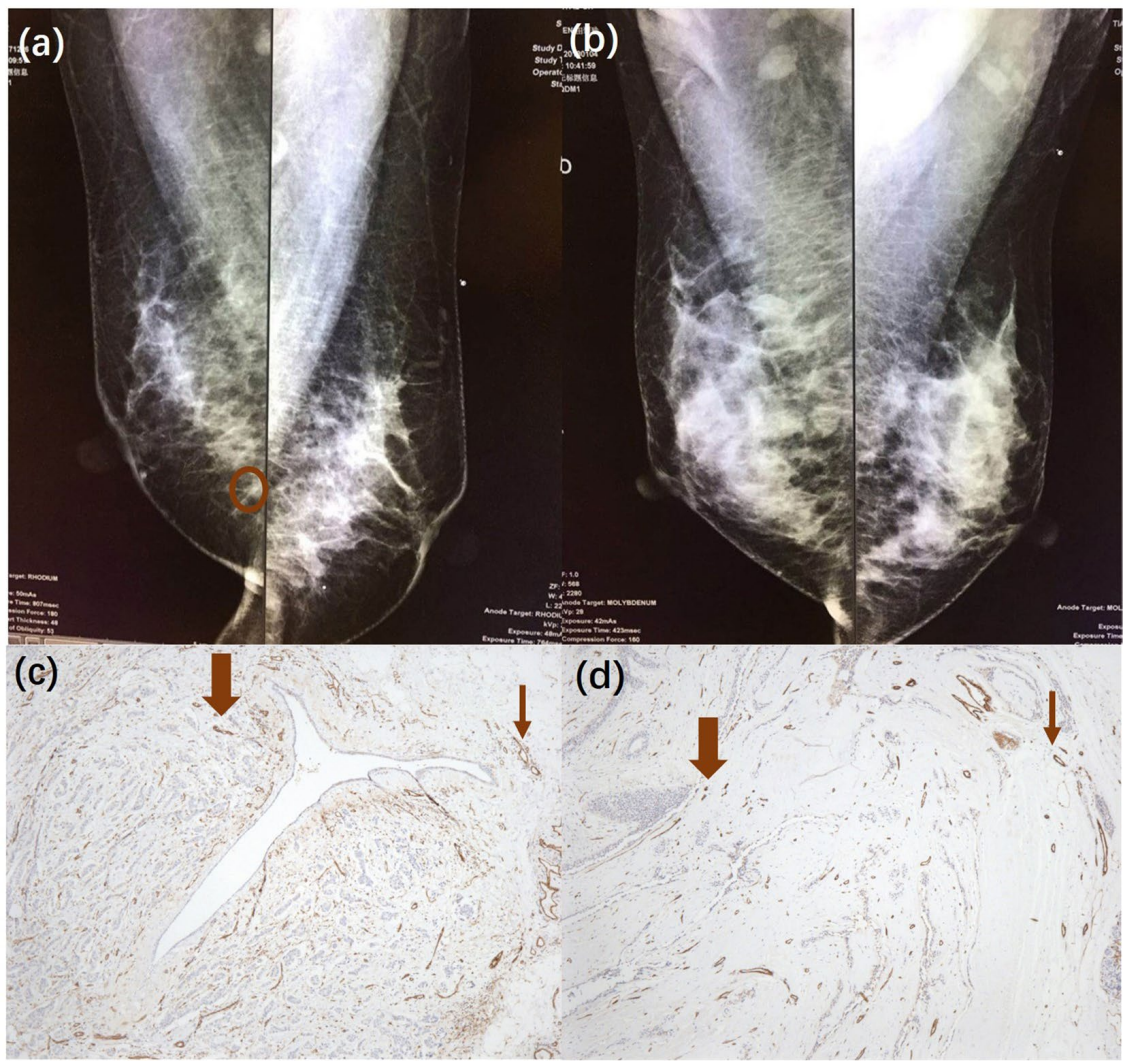
X-ray mammography results of the same (**a**) invasive breast cancer and (**d**) breast fibroadenoma shown in Fig. 3. CD31 immunochemistry (IHC) vascular staining results of the same (**c**) invasive breast cancer and (**d**) breast fibroadenoma shown in Fig. 3; both the tumour (thick brown arrow) and the surrounding area are depicted (thin brown arrow).

**Figure 5:**
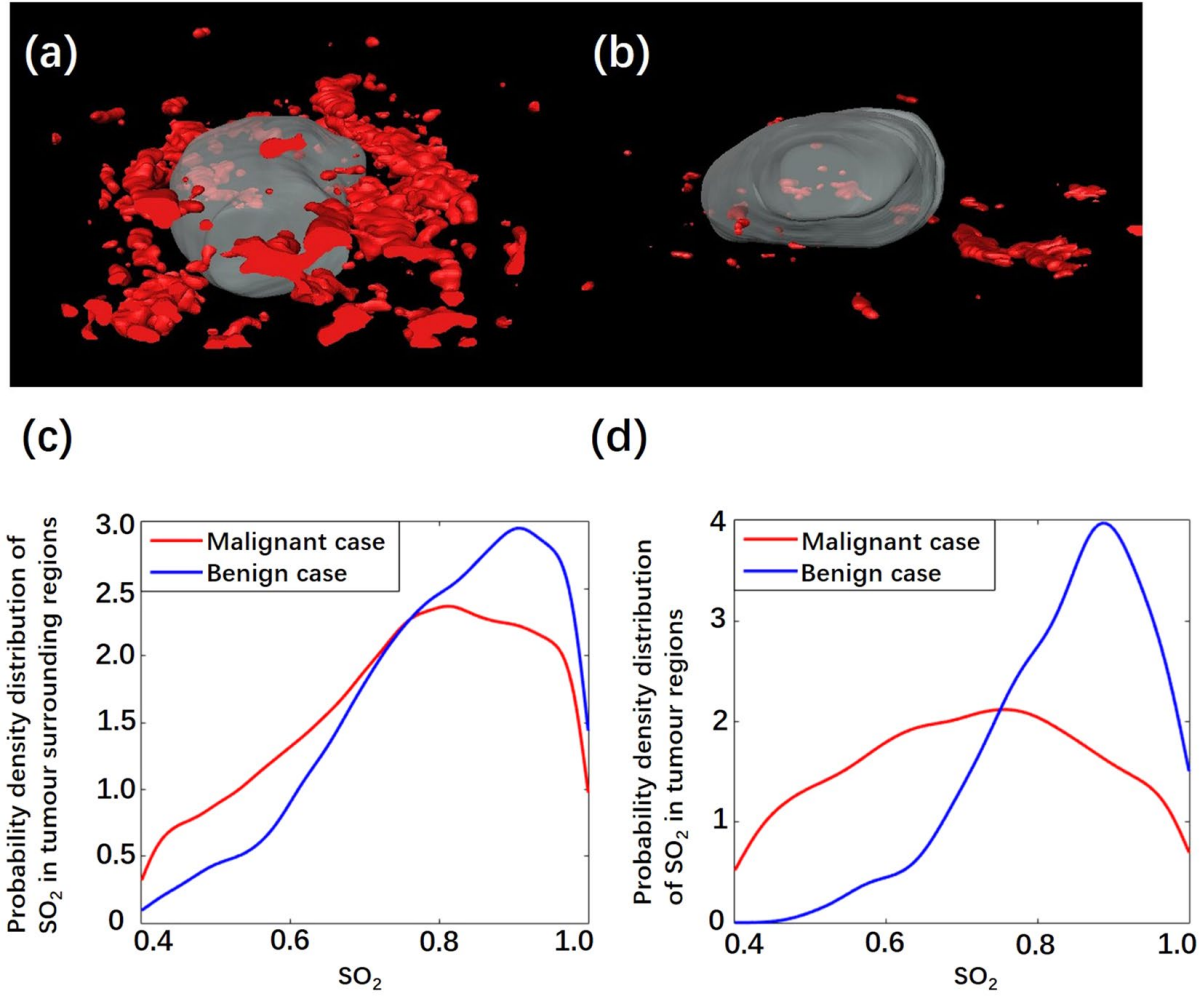
3D reconstruction vascular network maps of the same (**a**) invasive breast cancer and (**d**) breast fibroadenoma shown in Fig. 3. The SO_2_ density distribution in the (**c**) tumour regions and (**d**) tumour surrounding regions of these two tumours.

Examples of invasive breast cancers and benign breast lesions are provided in Figs. 3–5. Figure 3a,b depict the SO_2_/US fused imaging results of a malignant tumour (IBC) and a benign tumour (fibroadenoma), respectively. Color scale bars of SO_2_ are shown to the left of the imaging results. Lower SO_2_ values were observed in and surrounding the malignant tumour (Fig. 3a), which indicates a pattern differing from that of benign lesions (Fig. 3b). For this malignant case, confidently grading the malignant tumour based on the US result was difficult because the tumour had a clear margin and a regular shape, which is similar to benign tumours. In addition, X-ray mammography and CD31 immunochemistry (IHC) vascular staining results are shown in Fig. 4. From the X-ray results in Fig. 4a, the malignant tumour could hardly be detected since there was no obvious calcification and clear margin. From the X-ray results in Fig. 4b, the benign tumour could not be defined as in the US. From IHC vascular staining results, more CD31 vascular staining showed up in and around the malignant tumour (Fig. 4c) than the benign one (Fig. 4d), which is consistent with the 2D PA/US imaging results.

Figure 5a,b show the 3D vascular network maps of the same tumours shown in Fig. 3a,b. A more abundant vascular network showed up in regions surrounding the malignant tumour (see Supplementary Movie S2), while substantially less vascular network was shown around the benign tumour (see Supplementary Movie S3). The probability density distributions of SO_2_ in tumour and tumour surrounding regions are also displayed (Fig. 5c,d). An obviously low intra-tumoral SO_2_ distribution was detected in the malignant tumour compared to that in the benign tumour.

## Discussion

In this pilot clinical study, we presented 3D PA/US functional imaging on patients with small breast tumours (less than 2 cm). Based on the statistical results from 3D imaging data, invasive breast cancers showed obviously lower volumetric mean SO_2_ than both the benign group and the healthy group. With the SO_2_ cut-off value shown in the Statistical section, the sensitivity and specificity in differentiating T1 IBC and benign tumours were favourable. Conventional grey scale US has the advantage of clearly displaying the edge of breast tumours, even for some lesions that are not easily detected by X-ray, as shown previously in this study. With PA imaging added to grey scale US imaging, more functional information, such as haemoglobin imaging and SO_2_ distribution of the tumour, could potentially enhance the diagnostic confidence, especially in differentiating the malignant tumours from benign ones due to the difference in SO_2_ shown in PA/US dual mode imaging results.

Our result is consistent with a previous reported comparable clinical study in which more than 2000 breast tumours underwent handheld 2D PA/US functional imaging^13^. The results found that SO_2_ exhibited a lower trend in malignant tumours than in benign ones. In another clinical study based on photoacoustic mammography (PAM), the SO_2_ level in malignant tumours was also found to be lower than in the counterpart contralateral normal breast region of interest^17^, which was in agreement with the results of the present study.

The potential reasons for hypoxia in malignant tumours have been widely investigated^37–39^, and hypoxia likely arises as a result of an imbalance between the supply and consumption of oxygen. One predominant causative factor of the imbalance is severe structural and functional abnormalities in the tumour microcirculation, such as a disorganized vascular network, including dilations and elongated and tortuous shape. Interestingly, in addition to the significant differences in SO_2_ inside tumours, we also found a significant difference in SO_2_ in tumour surrounding regions. Although the mechanism underlying this difference is not clear yet, based on previous breast contrast enhanced imaging studies, a peripheral enhancement region of breast tumours is closely related to malignancy^40,41^. Histological features related to peripheral enhancement region include the existence of viable cancer cells^42^, expression of vascular endothelial growth factor (VEGF), and high microvascular density^41^, which may potentially cause a lower SO_2_ in this region. In addition to the significant difference in SO_2_ between benign and malignant tumours, we noticed that there is a weak trend (although not a significant difference) in which benign tumours have higher SO_2_ values than normal tissues. Currently we have no clear interpretation of the underlying mechanism, but a similar trend was also found in another study^43^. One potential explanation is that there are more abundant arteries in benign tumours than in normal tissues^44^, which could cause higher SO_2_ in benign tumours.

**Figure 6:**
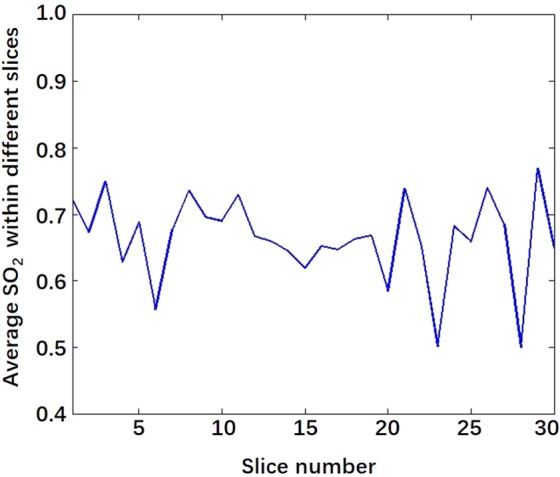
Average SO_2_ variation curve within different slices of a malignant tumour.

Compared to 2D functional PA/US imaging ^13,14^, 3D functional PA/US imaging is advantageous for several reasons. First, 3D PA/US imaging can provide quantified results based on 2D PA imaging, which may better present the overall functional imaging features of a tumour. To verify this, Fig. 6 displays the average SO_2_ values along different 2D slices within the tumour regions of a malignant case, showing that the SO_2_ values could largely vary from slice to slice, making it challenging to select an appropriate 2D slice as the representative image to calculate the SO_2_ for evaluation and diagnosis of the tumour. Second, compared to the scoring system employed in the previous 2D PA/US imaging study^13^, which is dependent on a manual scoring process, 3D quantification of the PAI physiological information may avoid intra-observer difference to a certain extent and thus deliver a diagnostic index with better repeatability and reliability. Third, 3D distribution of the vessel network could reveal more comprehensive spatial information on the tumoral vasculature than 2D distribution.

To date, there is no clear definition of tumour surrounding region. Some researchers defined thick echogenic rim surrounding some tumours as tumour surrounding regions^13^, while a thick echogenic rim doesn’t exist in every tumour, and it is hard to delineate the margin of the echogenic rim in some cases. In our study, we applied a consistent method to define the tumour surrounding regions (see Supplementary Fig. S3). Our method proved to be applicable to all tumours in our study, providing a repeatable and automatic analysis process without identifying tumour surrounding regions manually.

Our research does have some limitations. For instance, the “limited view” problem caused most of the reconstructed vessels to have an orientation that tended to be parallel with the scanning direction. However, our study implied that a valuable quantitative evaluation was still obtainable based on these “biased” reconstructed results. Besides, since it is hard to obtain an accurate optical fluence map, quantification of haemoglobin concentration is not currently available. Additionally, we noticed that after controlling the size distribution, there is a minor decrease in specificity when using SO_2_ in tumour surrounding regions as a biomarker to differentiate benign tumours from malignant tumours. One potential reason is that only two benign tumours with sizes from 1.6 cm to 2 cm is a small sample size. We also could not exclude the possibility that SO_2_ in tumour surrounding regions may be related to tumour size. The mechanism underlying this observation will be explored in the future with larger sample size, especially for benign tumours with larger tumour size. An increase of the overall sample size could also help further refine the cut-off value and improve the effectiveness of our quantification analysis. Therefore, although our analysis shows promising results for 3D PA/US functional imaging in terms of aiding the evaluation of breast tumours, both sensitivity and specificity should be further verified with a much larger sample size in the future.

In summary, 3D quantification analysis of oxygenation saturation level, especially in the peri-tumoral environment, shows promising results for the differentiation of benign tumours from malignant tumours in this study. Therefor 3D PA/US functional imaging demonstrates potential in the detection and diagnosis of breast tumours.

## Supplementary information

Supplementary Information.

Supplementary Table S1.

Supplementary Table S2.

Supplementary Movie S1.

Supplementary Movie S2.

Supplementary Movie S3.
