# Supplementary material for: Quantitative analysis of breast tumours aided by three-dimensional photoacoustic/ultrasound functional imaging
Source: Sci Rep. 2020 May 15;10:8047. doi: 10.1038/s41598-020-64966-6 (PMC7229157; doi:10.1038/s41598-020-64966-6)
Supplement: Supplementary file 1 — Supplementary Information. [file 41598_2020_64966_MOESM1_ESM.docx]

**Supplementary Information**

**Title:**

Quantitative analysis of breast tumours aided by three-dimensional photoacoustic/ultrasound functional imaging

**Authors:**

Meng Yang, ^1,5^ Lingyi Zhao,^2,5^ Fang Yang,^3^ Ming Wang,^1^ Na Su,^1^ Chenyang Zhao,^1^ Yang Gui,^1^ Yao Wei,^1^ Rui Zhang,^1^ Jianchu Li,^1^ Tao Han,^2^ Xujin He^3^, Lei Zhu^3^, Huanwen Wu,^4^ Changhui Li,^2,6^* Yuxin Jiang,^1^^,6^*

1 Department of Ultrasonography, Peking Union Medical College Hospital, Chinese Academy of

Medical Sciences & Peking Union Medical College, Beijing, China

2 Department of Biomedical Engineering, College of Engineering, Peking University, Beijing, China

3 Shenzhen Mindray Bio-Medical Electronics Co., Ltd., Shenzhen, China

4 Department of Pathology, Molecular Pathology Research Center, Peking Union Medical College Hospital, Chinese Academy of Medical Science, Beijing, China

5 These authors contributed equally to this work

6 These authors contributed equally to this work

*Corresponding information:

Yuxin Jiang

Email: [yuxinjiangxh@163.com](mailto:yuxinjiangxh@163.com)

Address: Shuaifuyuan No.1, Dongcheng District, Beijing, 100730, China

Tel: 010-69155491

Changhui Li

Email: [chli@pku.edu.cn](mailto:chli@pku.edu.cn)

Address: Yiheyuan Road No.5, Haidian District, Beijing, 100871, China

Tel: 010-62767894

**Supplementary Figure S1:**


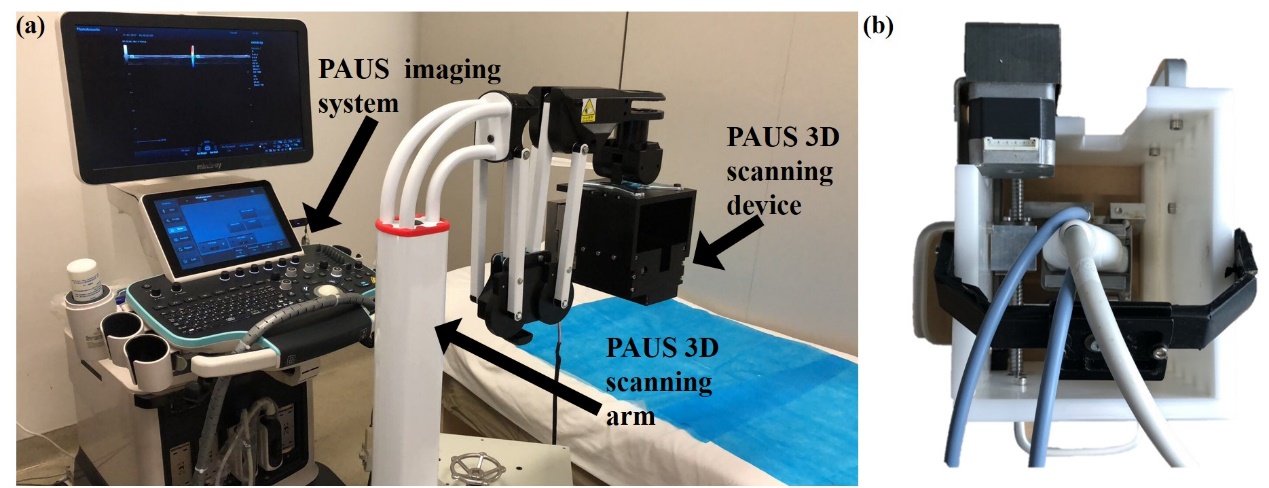


Supplementary Figure S1. (a) 2D and 3D PA/US dual mode functional imaging system. (b) Top view of the PA/US 3D scanning device

**Supplementary Figure S2:**


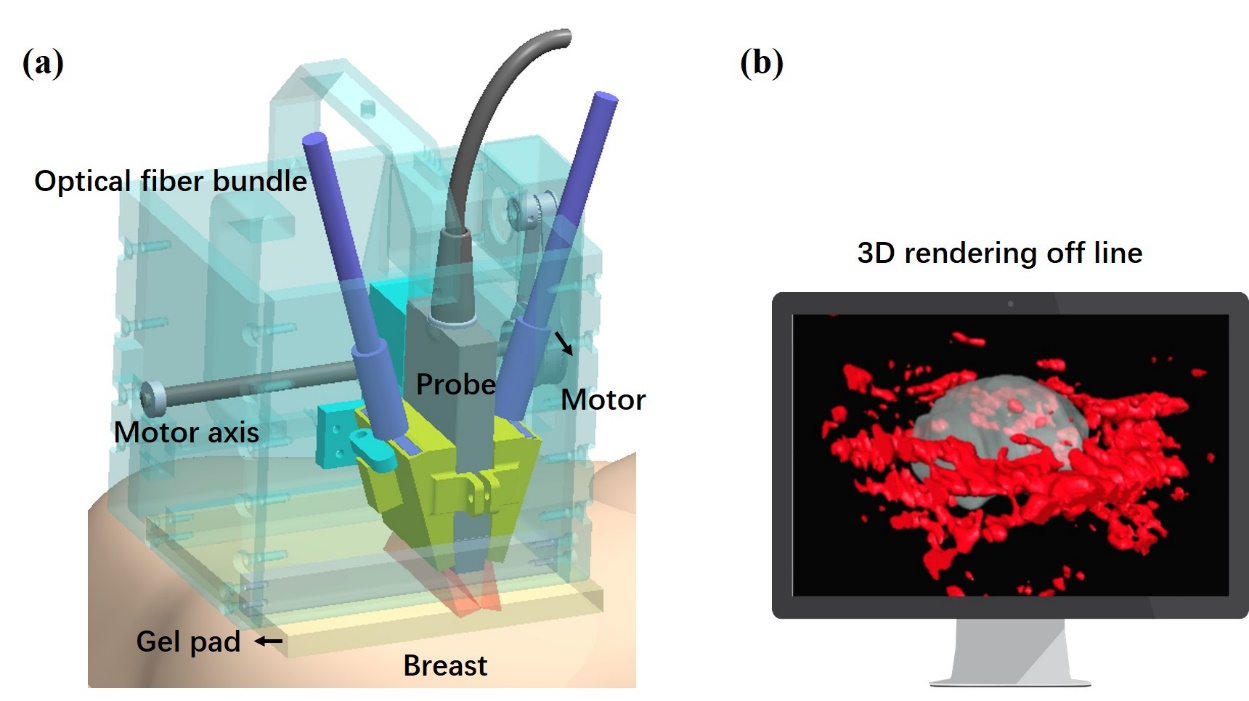


Supplementary Figure S2. Representations of (a) the PA/US 3D scanning device and (b) 3D rendering offline

**Supplementary Figure S3:**


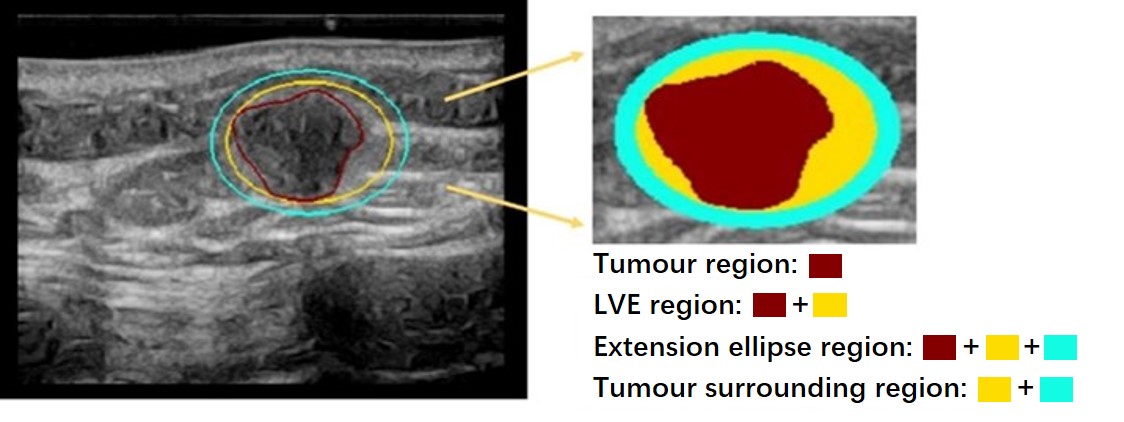


Supplementary Figure S3. Representation for definitions of the tumour region and the tumour surrounding region

**Supplementary Table S1.**

Detailed information of 24 studied breast tumours

**Supplementary Table S2.**

Tumour size and correspondent sample size in each subgroup analysis

**Supplementary Movie S1.**

3D scanning animation of the PA/US 3D imaging system

**Supplementary Movie S2.**

3D vascular network maps of the same malignant tumour shown in Fig. 3a.

**Supplementary Movie S3.**

3D vascular network maps of the same benign tumour shown in Fig. 3b.
